# Safety and efficiency of SGLT2 inhibitor combining with insulin in subjects with diabetes: Systematic review and meta-analysis of randomized controlled trials

**DOI:** 10.1097/MD.0000000000006944

**Published:** 2017-05-26

**Authors:** Yingying Yang, Shi Chen, Hui Pan, Yun Zou, Bo Wang, Guixia Wang, Huijuan Zhu

**Affiliations:** a Department of Endocrinology, Endocrine Key Laboratory of Ministry of Health, Peking Union Medical College Hospital, China Academy of Medical Sciences Peking Union Medical College and Peking Union Medical College, Beijing; b Department of Orthopedics, The Second Hospital of Jilin University, Changchun, Jilin; c Health Science Popularization Research Center, Chinese Academy of Medical Sciences, Beijing; d Department of Endocrinology and Metabolism, The First Hospital of Jilin University, Changchun, Jilin, China.

**Keywords:** FPG, genital tract infection, HbA1c, hypoglycemia, insulin, insulin dose, meta-analysis, SGLT2 inhibitors, urinary tract infection

## Abstract

**Background:**

We aimed to assess the safety and efficiency of the novel sodium glucose co-transporter 2 (SGLT2) inhibitor in combinations with insulin for type 1 and type 2 diabetes mellitus (T1DM and T2DM).

**Methods:**

We searched Medline, Pubmed, Embase, and the Cochrane Collaboration Library from January 2010 to December 2016 without restriction of language. FDA data and Clinical Trials (http://www.clinicaltrials.gov) were also searched. Study selection, data extraction, and evaluation of risk of bias were performed by 2 persons independently. The risk of bias was assessed by Cochrance System Evaluate Method and Q test was used to evaluate the heterogeneity between studies. We used random effect model to analyze the results by Revman 5.3. This meta-analysis has been registered at online public registry PROSPERO (registration number is: CRD42017054718).

**Results:**

Nine trials including 3069 patients were analyzed. Compared with control group, SGLT2 inhibitor produced absolute reduction in glycosylated hemoglobin A1c (HbA1c) (MD −1.35%, 95% confidence interval [CI] [−2.36 to −0.34], *P* = .009), fasting plasma glucose (FPG) (MD −1.01 mmol/L, 95%CI [−1.98 to 0.04], *P* = .04), insulin dosage (MD −4.85 U/24 hours, 95%CI [−7.42 to −2.29], *P* = .002), and body weight (MD −2.30 kg, 95%CI [−3.09 to −1.50], *P* < .00001). But the risk of hypoglycemia (OR 1.18, 95%CI [0.86, 1.61], *P* = . 30) and urinary tract infection (UTI) (OR 1.34, 95%CI [0.79, 2.27], *P* = .28) were proved as no difference and genital tract infection (GTI) with SGLT2 inhibitors was higher than control group (OR 2.96, 95%CI [1.05, 8.37], *P* = .04), in which cases were mild and responded to the therapy. According to the subgroup analysis, SGLT2 inhibitors had a similar effect in effective factors of both T1DM and T2DM, but the risk of GTI mainly increased in T2DM versus T1DM (T1DM OR 0.27 [0.01, 7.19], *P* = .43 vs T2DM OR 4.28 [2.00, 9.16], *P* = .0002).

**Conclusion:**

SGLT2 inhibitors have improved the HbA1c, FPG, and body weight when combined with insulin and decreased the dose of insulin without increasing the risk of hypoglycemia. However, SGLT2 inhibitor was proved to be related to the events of GTI, despite SGLT2 inhibitors appeared to be well tolerated. We suggest that more monitoring should be done to prevent the events of GTI, and more randomized controlled trials should be planned next step.

## 1 Introduction

Early initiation of basal insulin has been used not only in type 1 diabetes but also in type 2 diabetes; nevertheless, some patients will develop insulin resistance, and higher doses of insulin may be required to lower the blood glucose which can lead to weight gain and the risk of hypoglycemia. Therefore, there are many different oral antidiabetic drugs (OADs) approved in DM combined with insulin such as metformin, sulfonylureas, α-glucosdase inhibitor, dipeptidyl peptidase-4 inhibitors (DDP-4 inhibitor), GLP-1 receptor agonist, and thiazolidinedione, but they all have some defects despite the fact of improving the insulin resistance and blood glucose level, for example, metformin has gastrointestinal effect that even cannot be tolerated by some patients; thiazolidinedione has a risk of bladder cancer, heart failure, and change in bone density which may affect its longer duration of use; GLP-1 agonists will need additional injections and higher in cost. So, it is necessary to explore a new agent used in combination with insulin that will produce the reductions in glycosylated hemoglobin A1c (HbA1c), body weight, insulin requirements, and incidences of hypoglycemia with small side effect in DM.^[1]^ Sodium glucose co-transporter 2 (SGLT2) inhibitor is a new kind of oral antihyperglycemic drug which was approved by Food and Drug Administration (FDA) on March, 2013 available in type 2 diabetes mellitus (T2DM).^[2,3]^ SGLT2 expressed in the proximal renal tubules accounts for about 90% of the reabsorption of glucose from tubular fluid, so that it can block the reabsorption of glucose by the kidney, increasing glucose excretion, and reducing blood glucose levels in people with diabetes who have elevated blood glucose levels.^[4]^ SGLT2 inhibitors including dapagliflozin, empagliflozin, capagliflozin, and tofogliflozin have been only applied in the T2DM. But many large randomized trails are under progress for the prospect of possibilities of SGLT2 inhibitors of add-on therapy to insulin in type 1 diabetes mellitus (T1DM). Previous studies indicated that SGLT2 inhibitors could lower glycemic level either as monotherapy or add-therapy on insulin and other antihyperglycemic drug in DM.^[5–8]^ We are interested in whether SGLT2 inhibitor will be safe and efficient enough as add-on therapy on insulin due to its noninsulin dependent mechanism. SGLT2 inhibitors are considered to link to an increased incidence of diabetic ketoacidosis (DKA) in T2DM recently,^[9]^ particularly in those patients treated with insulin. Other adverse outcome includes genital tract infections (GTIs) and, to a lesser extent, urinary tract infections (UTIs), which may limit their utility in some patients.^[10]^ So far no meta-analysis has evaluated the safety and efficiency in combination of SGLT2 inhibitors and insulin. So, we aim to evaluate the safety and efficacy of SGLT2 inhibitors combined with insulin in DM.

## 2 Methods

### 2.1 Inclusion criteria

#### 2.1.1 The type of study

We included all studies which were randomized or quasi-randomized controlled trials; we excluded case report, case review, nonrandomized controlled trials. All analyses were based on previous published studies; thus, no ethical approval and patient consent are required.

#### 2.1.2 The type of participates

Patients aged more than 18 years old, HbA1c between 7% and 12%, treated by insulin who were diabetes mellitus diagnosed by WHO diagnostic criteria.

#### 2.1.3 Types of intervention

We included studies comparing SGLT2 inhibitors plus insulin versus placebo plus insulin or only insulin no matter the dose and kind of SGLT2 inhibitor and insulin; we excluded studies in which SGLT2 inhibitors as a monotherapy in experimental group, the insulin therapy could be multiple daily injections consisting of long-acting (basal) plus short-acting insulin or continuous subcutaneous insulin infusion pump.

#### 2.1.4 Type of comparisons

The compare group was placebo combined with insulin or only insulin in which the insulin method was same to experimental group. We excluded the studies in which the compare group had no therapy or placebo.

#### 2.1.5 Types of outcomes

We included studies that primary outcomes should include one of following outcomes: adverse reactions including hypoglycemia, UTI, and GTI, the effective indicators including HbA1c and fasting plasma glucose (FPG).

### 2.2 Search strategy

We searched Medline, Pubmed, Embase, and the Cochrane Collaboration Library from January 2010 to December 2016, without restriction of country, race, language, or publication year. FDA data and Clinical Trials (http://www.clinicaltrials.gov) were also searched. Our search strategy used the following terms: “sodium-glucose cotransporter 2 inhibitor”, “SGLT2 inhibitor”, “canagliflozin”, “dapagliflozin”, “empagliflozin”, “sapagniflozin”, “ipragliflozin”, “luseogliflozin”, “tofogliflozin”, “T1DM”, “T2DM”, “type 1 diabetes”, “type 2 diabetes”, and “Diabetes mellitus”. These terms were adjusted to comply with the relevant rules in each database.

### 2.3 Selection of eligible studies

First of all, randomised or quasi-randomised controlled trials were identified through title or abstract (if necessary). Further, based on inclusion and exclusion criteria, eligible studies were included through abstract or full text (if necessary). This was performed by 2 reviewers (YY and YZ) independently. Discrepancies were resolved by discussion between the 2 reviewers, and unresolved disagreement was referred to a 3rd reviewer (SC).

### 2.4 Risk of bias

Two reviewers (YY and YZ) independently applied the Cochrane risk of bias tool to assess the risk of bias of randomized trials, including random sequence generation, allocation concealment, blinding, incomplete data regarding outcome, selective reporting, and other items (ie, groups comparable at baseline, funder, and incomplete information in the text).

### 2.5 Data synthesis and analysis

All outcomes were pooled using RevMan5.3 software. For continuous and discontinuous data, differences were calculated using mean and OR, respectively. All results were estimated from each study with 95% confidence interval (CI). Heterogeneity was assessed using the chi-square test and the *I*^2^ statistics, the random-effect model was adopted regardless of *I*^2^. If our primary outcome data (ie, standard deviation and variance measures) were missing or incomplete, we emailed the corresponding authors or the sponsors. When necessary, the value of standard deviation was calculated from CI or standard error as described in the Cochrane Handbook.

### 2.6 Subgroup analyses

Our prior hypotheses to explain potential heterogeneity across studies included: T1DM and T2DM involve different etiologies, pathogenesis, and clinical manifestations; treatment interventions with SGLT2 inhibitors, insulin, and other oral hypoglycemic drugs vary from different studies; study quality (loss to follow-up and blinding status) also differs. To explore these hypotheses, we estimated the differences in treatment effects between subgroups or treatment–subgroup interaction.

## 3 Results

### 3.1 Characteristics of included studies

**Figure 1:**
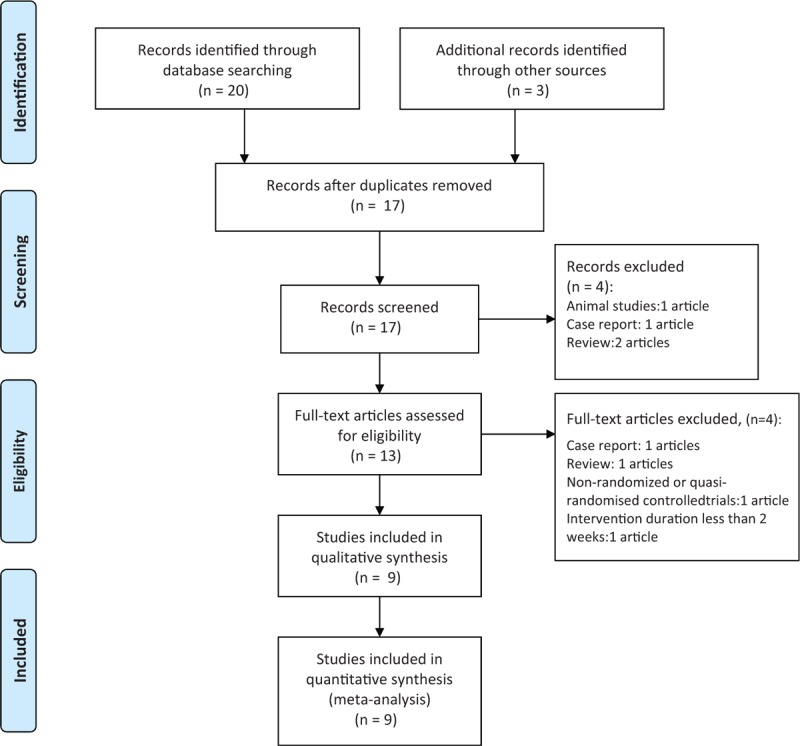
Selection of eligible studies.

We found 17 articles after comprehensive searching which were all in English. According to the inclusion and exclusion criteria, we eliminated 8 studies. During the process of studies selection, 1 of them was animal study, 1 of them were case reports, 3 of them were reviewes, 1 of them were not randomized or quasi-randomized controlled trials, intervention duration of 1 study was less than 2 weeks. Finally, we included 9 studies.^[8,11–18]^ (Fig. 1 shows the selection of eligible studies).

Eight of 9 studies were double-blinded, randomized, placebo-controlled trials, 1 study was randomized noplacebo control trail, 2 studies were IIa pilot trials; 1 study was single-center, 6 studies were multicenter; participates aged between 18 and 65 years old; HbA1c was between 7% and 12%; BMI of the most patients were more than 24 kg/m^2^; the experimental group in 9 studies were SGLT2 inhibitor combined with insulin, SGLT2 inhibitors were respectively dapagliflozin (3 studies), empagliflozin (3 studies), sapagniflozin (1 study), canagliflozin (1 study), and tofogliflozin (1 study), while the control group were placebo with insulin or insulin itself. Insulin therapy was not limited as long as the insulin therapy was the same in both 2 groups. Intervention duration ranged from 2 to 78 weeks. (Table 1 shows the characteristic of 8 included studies).

**Table 1:**
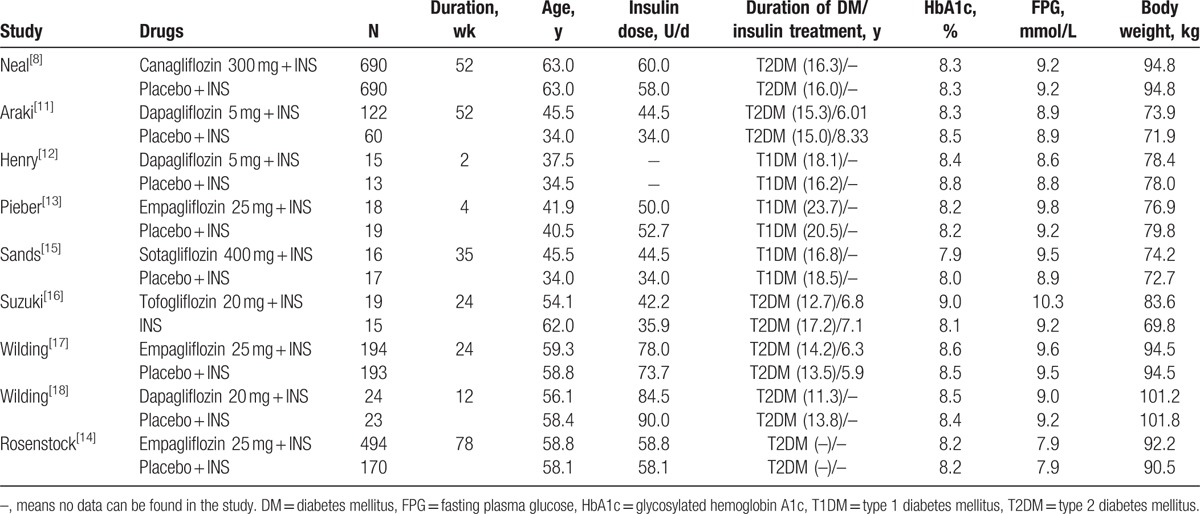
Characteristic of 9 included studies.

According to the Cochrane Risk of Bias tool, we assessed the quality of the included studies in 8 aspects: random assignment method, allocation concealment, blinding, reliable outcome measure, and other source of bias. As a result, these studies had low risk of bias. (Table 2 shows the assessment of the risk of bias in 8 included studies).

**Table 2:**
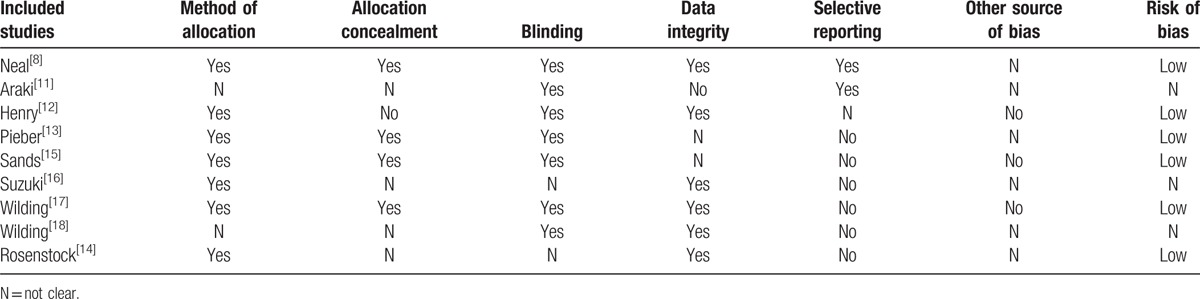
Assessment of the risk of bias in 9 included studies.

### 3.2 Efficiency

**Figure 2:**
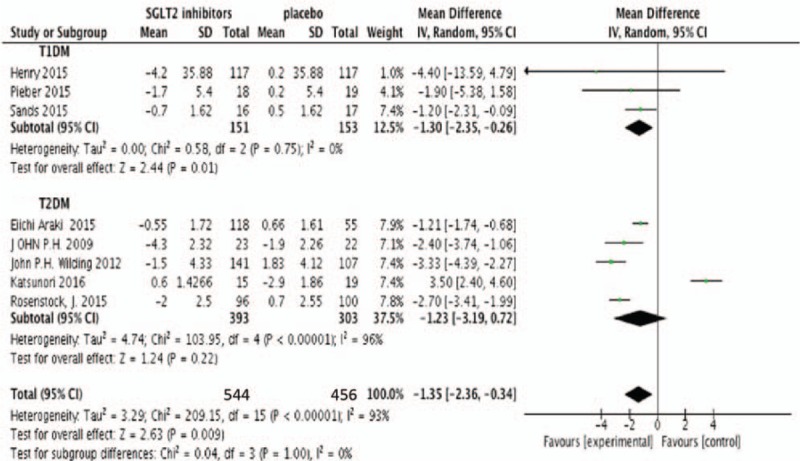
Random-effects meta-analysis of the effects of SGLT2 inhibitor versus placebo on change from baseline of HbA1c. Vertical lines represent no treatment effect. Squares and horizontal lines represent the point estimates and associated confidence interval for each comparison, respectively. Point estimates to the left of the vertical line reflect HbA1c reduction of SGLT2 inhibitors favoring the intervention rather than the control. Results expressed in standard deviation units (effect size). HbA1c = glycosylated hemoglobin A1c, SGLT2 = sodium glucose co-transporter 2.

**Figure 3:**
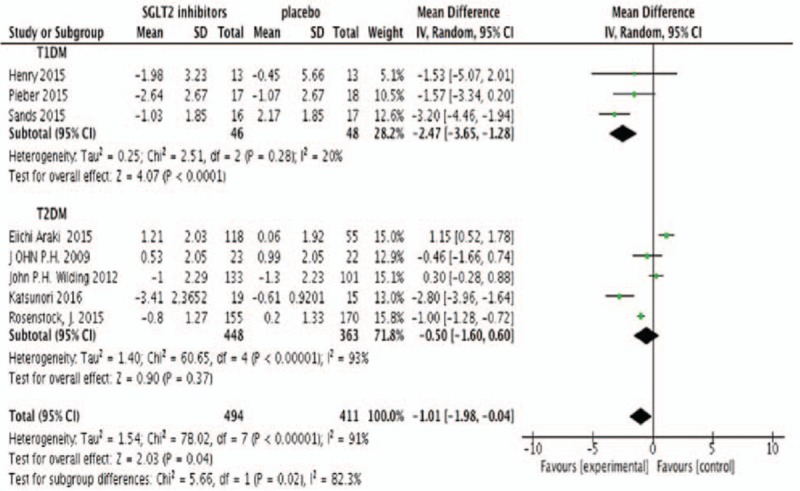
Random-effects meta-analysis of the effects of SGLT2 inhibitor versus placebo on change from baseline of FPG. FPG = fasting plasma glucose, SGLT2 = sodium glucose co-transporter 2.

**Figure 4:**
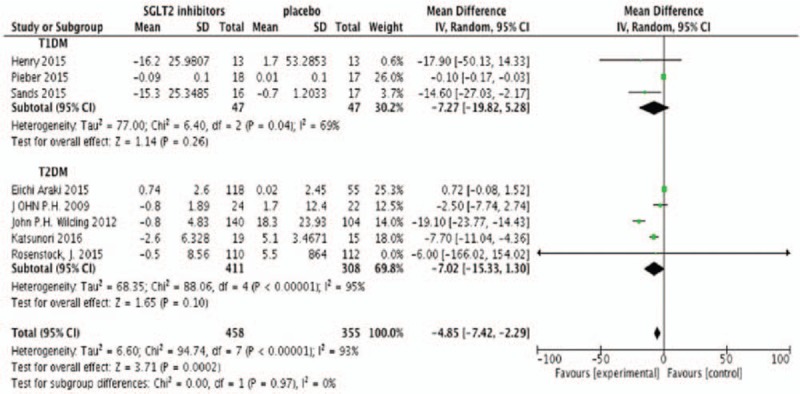
Random-effects meta-analysis of the effects of sodium glucose co-transporter 2 (SGLT2) inhibitor versus placebo on change from baseline of insulin dose.

**Figure 5:**
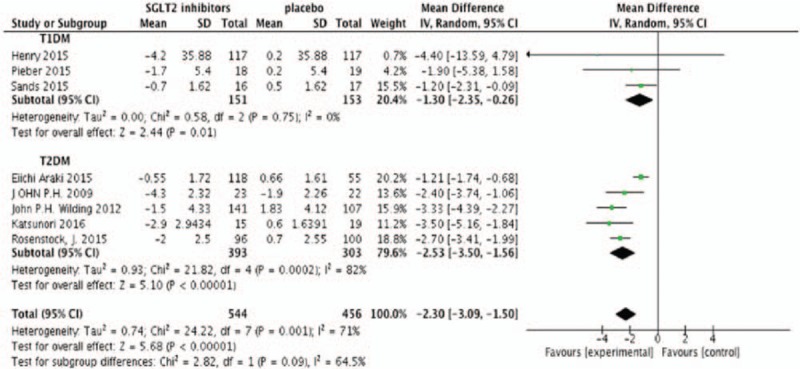
Random-effects meta-analysis of the effects of sodium glucose co-transporter 2 (SGLT2) inhibitor versus placebo on change from baseline of body weight.

Eight studies were included in the meta-analysis with efficiency parameters (Neal's study did not provide the change from baseline on outcomes, but change from placebo). All meta-analyses were performed by the random effect model no matter the heterogeneity among studies. Meta-analysis found that SGLT2 inhibitors produced absolute reductions in change from baseline of HbA1c as add-on treatment compared with placebo (N = 1000, MD −1.35%, 95%CI [−2.36 to −0.34], *P* = .009) (Fig. 2). SGLT2 inhibitors also determined a modest but statistically significant decrease in FPG (N = 905, MD −1.01 mmol/L, 95%CI [−1.98 to 0.04], *P* = .04) (Fig. 3). Figure 4 showed the effects of SGLT2 inhibitor versus placebo on insulin doses. In placebo-controlled trials, SGLT2 inhibitors decreased the insulin dosage (N = 813, MD −4.85 U/24 hours, 95%CI [−7.42 to −2.29], *P* = .002) (Fig. 4). Figure 5 showed the effects of SGLT2 inhibitor versus placebo on body weight. The body weight reduction was 2.3 kg with SGLT2 inhibitors as add-on treatment in DM compared with placebo (N = 1000, MD −2.30 kg, 95%CI [−3.09 to −1.50], *P* < .00001 (Fig. 5).

Subgroup analysis showed that the type of DM was associated with a moderate treatment effect in FPG (χ^2^ = 5.66, *P* = .02, *I*^2^ = 82.3%). There was also no significant subgroup interaction in HbA1c and body weight. Heterogeneity among studies in T2DM were higher than T1DM with HbA1c (96% vs 0%), FPG (93% vs 20%), insulin dose (69.8% vs 30.2%), and body weight (96% vs 0%). SGLT2 inhibitors had a similar effect in HbA1C (T1DM −1.30% [−2.35, −0.26] vs T2DM −1.23% [−3.19, −0.72]), FPG (T1DM −2.47 mmol/L [−3.65, −1.28] vs T2DM −0.50 mmol/L [1.60, 0.60]), insulin dose (T1DM −7.27 U/24 hours [−19.82, 5.28] vs T2DM −7.02 U/24 hours [−15.33, 1.30]), and body weight (T1DM −1.30 kg [−2.35, −0.26] vs T2DM −2.53 kg [−3.10, −1.56]) with T1DM and T2DM.

### 3.3 Safety

#### 3.3.1 Risk of GTI

**Figure 6:**
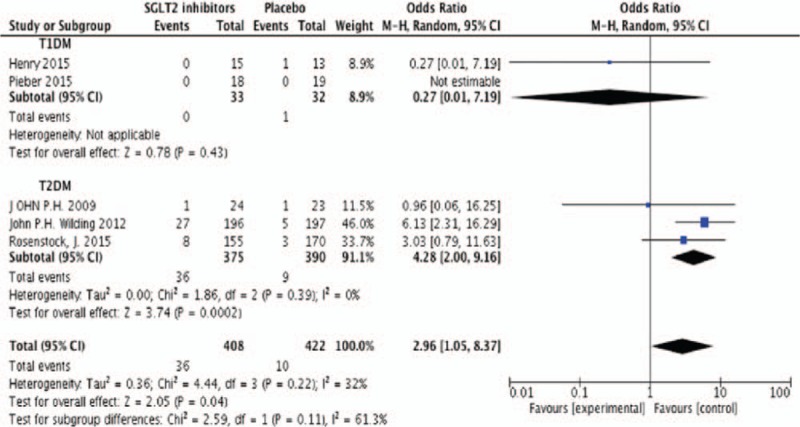
Random-effects meta-analysis of the effects of SGLT2 inhibitor versus placebo on events of GTI. GTI = genital tract infection, SGLT2 = sodium glucose co-transporter 2.

Six studies were included in the meta-analysis with 430 participates. Random effects model was used in meta-analysis. Events of GTI were higher in SGLT2 inhibitors group compared with control group (OR 2.96, 95%CI [1.05, 8.37], *P* = .04) (Fig. 6). However, most events were classified as mild or moderate in intensity and responded to therapy. Among studies included in our research, GTI led to discontinuation of 2 patients in Wilding study.^[17]^

#### 3.3.2 Risk of hypoglycemia and UTI

**Figure 7:**
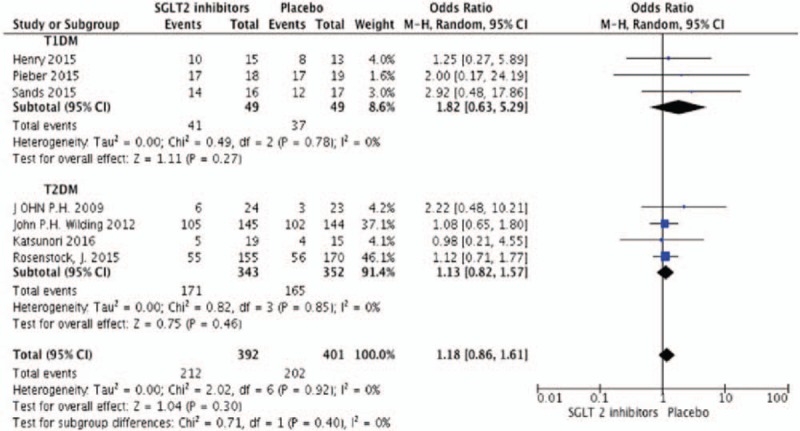
Random-effects meta-analysis of the effects of sodium glucose co-transporter 2 (SGLT2) inhibitor versus placebo on events of hypoglycemia.

**Figure 8:**
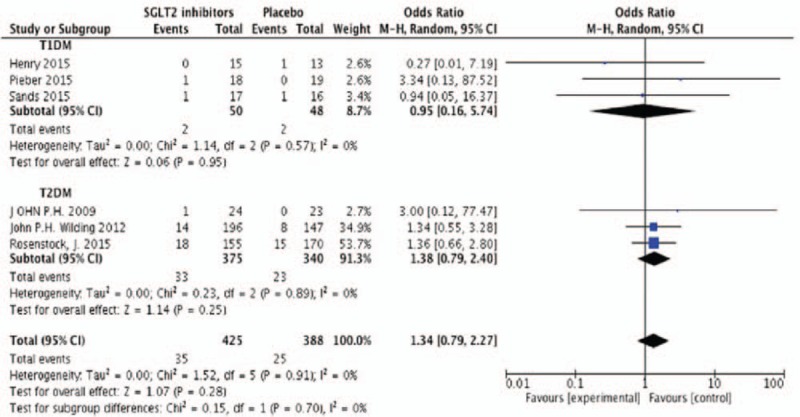
Random-effects meta-analysis of the effects of SGLT2 inhibitor versus placebo on events of UTI. SGLT2 = sodium glucose co-transporter 2, UTI = urinary tract infection.

According to the diagnostic criteria, hypoglycemia in DM means glucose level less than 3.9 mmol/L (American Diabetes Association, ADA and The Endocrine Society).^[19]^ Seven studies were included in the meta-analysis with 571 participates. There was no statistical difference in the incidence of hypoglycemia (but a tendency to increase) (OR 1.18, 95%CI [0.86, 1.61], *P* = .3) (Fig. 7) and urinary infection (OR 1.34, 95%CI [0.79, 2.27], *P* = .28) (Fig. 8) compared to placebo by random effects model. Araki et al^[11]^ reported that the incidence of mild hypoglycemia was higher in dapagliflozin group (19.5% vs 23.3% in the placebo group), but dapagliflozin was still safe under proper management. During the study of Wilding et al,^[18]^ 3 patients experienced severe adverse effect due to hypoglycemia (2 receiving dapagliflozin 5 mg and 1 receiving placebo) but no patient discontinued because of this adverse incidence. Pieber et al^[20]^ reported a patient discontinued study participation due to hypoglycemia. There were only 2 patients receiving dapagliflozin discontinued the study because of lower UTI.

## 4 Discussion

Insulin therapy plays a crucial part in DM. Many patients have achieved their normal HbA1c depending on the replacement therapy of insulin. Large doses of insulin will lead to the increased weight and the event of hypoglycemia.^[21]^ Previous research founded that even large doses of insulin could not improve the insulin resistance in obese patients with diabetes. Instead, some agents that are available to be added to the increasing insulin dosage also have a potential to increase insulin-related hypoglycemia and weight gain.^[22]^ Therefore, we need an agent that can work independent of the β-cell, reduce HbA1c effectively without provoking hypoglycemia, and additionally counter weight gain arising out of progressive increase in insulin dose. SGLT2 inhibitors become promising as a useful addition to the current therapeutic options in either T1DM or T2DM. It decreases blood glucose by increasing reabsorption of glucose from tubular fluid in insulin-independent mechanism.^[23]^

In our systematic review, we evaluated the safety and efficiency when SGLT2 inhibitor was used as insulin add-on therapy in diabetes mellitus. Nine studies were included in our research, Cochrane risk bias tool was used to evaluate the risk of bias, all of which had a low risk of bias and thus increased the reliability of the results. Three limitations merit consideration. First, durations of some included studies were short, study period was insufficient, only 1 study evaluated the long-term efficiency (including the clinical changes in laboratory values of interest) and safety of SGLT2 inhibitors (including the event of malignant neoplasm); thus, longer duration of observation is needed to understand the long-term benefits and risks of SGLT2 inhibitors. Second, heterogeneity among studies in T2DM were higher than 20% when evaluating the efficiency of SGLT2 inhibitors, which influenced the final heterogeneity in DM. However, the source of heterogeneity was still not clear due to the small number of study. Finally, all studies we included were in English and published the positive result which may lead to systematic error.^[24]^

SGLT2 inhibitors produced a significant reduction of HbA1c and FPG levels in our study which were consistent with the results reported in previous meta-analyses.^[25–29]^ Wilding et al^[17]^ reported the improvement of glycemic outcome were dose-independent,^[17]^ the study of Araki et al^[11]^ showed a more remarkable effect of SGLT2 inhibitors on glycemic improvement in East Asian patients who had already been treated with insulin, especially in the Japanese population with smaller BMI and lower doses of insulin. This glycemic control was achieved without an increase in mean daily insulin requirement, which will lead to weight gain and insulin resistance. So, there was significant weight loss in SGLT2 inhibitor group. Rosenstock et al^[14]^ thought it was due to urinary glucose excretion and mild osmotic diuresis.^[14]^ The body weight reduction was higher in T2DM than in T1DM. It is not surprizing that patients in T2DM were fatter with metabolism syndrome, and their requirements of insulin were higher than T1DM because of the insulin resistance which will lead to more weight gain. This meaningful result indicated that the therapy of SGLT2 inhibitor may be a better accompanying OADs with insulin therapy. It was known that patients treated with insulin were at a higher risk of hypoglycemia, which can increase macrovascular events and mortality.^[30,31]^ The results of our study finally indicated that SGLT2 inhibitors achieved glycemic control without increasing the incidence of hypoglycemia. We speculate that the reason is that it can work mainly by renal way but not the islet cell like others B-cell independent OADs such as GLP-1 receptor agonists and DPP-4 inhibitors are not related to the risk of hypoglycemia when combining with insulin.^[32]^ Patients with diabetes are at an increased risk of infections, Monami et al^[33]^ reported that genital and urinary infections rather frequent but usually mild in SGLT2 inhibitors group that appeared to be well tolerated. Our meta-analysis demonstrated that GTI occurred more often in SGLT2 inhibitors compared to placebo while UTI has a small increase in SGLT2 groups but with no statistical significance. In addition, it seemed that GTI occurred more frequently in T2DM with SGLT2 inhibitors plus insulin therapy compared with T1DM. Indeed, diabetes is considered as a risk factor for UTI and GTI, particularly in the setting of uncontrolled hyperglycemia.^[34]^ In fact, use of the 3 drugs (canagliflozin, dapagliflozin, and empagliflozin) is accompanied by increased genital infections compared with a placebo and affects more in women than men (by 4–5 times), mostly as vulvitis. In women, the diagnosis is mostly mycoticvulvovaginitis, and in men, mycoticbalanitis. In clinical trials, the incidence of genital infections with the maximum drug dosage is between 5% and 15%, and is not proportional to the amount of glycosuria, and thus, not related to SGLT2 doses.^[35]^ However, they failed to demonstrate a definitive dose relationship between glycosuria and GTI. Most genital infections appeared to occur within the first 24 to 26 weeks of SGLT2 inhibitor treatment, and the glycosuria associated with SGLT2 inhibitor treatment were likely contributed to the increased risk of these infections. But cases in our study were generally mild and responded to standard therapy.^[36]^

Our study did not perform meta-analysis in the incidence of DKA due to the limited number of studies (only Henry's and Sand's study). About 4.3% and 6.0% of patients had DKA during the treatment of canagliflozin 100 and 300 mg versus placebo,^[12]^ while 2 of 16 patients reported DKA in sotagliflozin plus insulin group compared with no one in placebo group.^[15]^ Many international administrations declared that SGLT2 inhibitors may lead to DKA, especially euglycemic DKA. It is widely known that patients with deficient insulin are prone to DKA, which is often triggered by some specific occasions like insulin dose reduction and other severe diseases. But SGLT2 inhibitor-related DKA may have a different pathogenesis compared to classic DKA. It often occurred with a slightly elevated or even normal plasma glucose level which is hard to discover. Asymptomatic rises in β-hydroxybutyrate was found in many SGLT2 inhibitor clinical trials.^[37]^ Researchers tended to explain this unexpected symptoms by the imbalance between glucose production inside body and renal glucose clearance. Now SGLT2 inhibitors have not been approved for use in T1DM, except in clinical trials, one of the reason is that patients with T1DM are at a higher risk of DKA. So, some experts recommend that SGLT2 inhibitors should only be considered as add-on therapy in T2DM until their insulin dose become stabilized and it is also important to monitor the blood ketone when changing insulin dose.^[38]^

In summary, SGLT2 inhibitor was effective in diabetes mellitus as add-on to insulin therapy. It improved HbA1c, FPG, and body weight at a relatively decrease in insulin dose without remarkably risk of hypoglycemia. However, the events of GTI increased in SGLT2 inhibitor group, especially in T2DM, despite it proved to be mild and tolerated. Our research indicated that SGLT2 inhibitor is good enough on controlling glycemic level but more attention should be paid on the risk of genital infection. Furthermore, more large, multicenter trails need to be performed to discuss the safety of SGLT2 inhibitors combined with insulin in subjects with DM.
