# Safety and efficiency of SGLT2 inhibitor combining with insulin in subjects with diabetes: Systematic review and meta-analysis of randomized controlled trials: Erratum

**DOI:** 10.1097/MD.0000000000007458

**Published:** 2017-06-30

In the article, “Safety and efficiency of SGLT2 inhibitor combining with insulin in subjects with diabetes: Systematic review and meta-analysis of randomized controlled trials”,^[1]^ which appeared in Volume 96, Issue 21 of *Medicine*, “Yingying Yang and Shi Chen contributed equally to this paper” should have appeared in the footnote.
